# Plasma Proprotein Convertase Subtilisin/Kexin Type 9 (PCSK9) as a Possible Biomarker for Severe COVID-19

**DOI:** 10.3390/v15071511

**Published:** 2023-07-06

**Authors:** Patricia Mester, Pablo Amend, Stephan Schmid, Martina Müller, Christa Buechler, Vlad Pavel

**Affiliations:** Department of Internal Medicine I, Gastroenterology, Hepatology, Endocrinology, Rheumatology and Infectious Diseases, University Hospital Regensburg, 93053 Regensburg, Germany; patricia.mester@klinik.uni-regensburg.de (P.M.); pablo.amend@stud.uni-regensburg.de (P.A.); stephan.schmid@klinik.uni-regensburg.de (S.S.); martina.mueller-schilling@klinik.uni-regensburg.de (M.M.); vlad.pavel@klinik.uni-regensburg.de (V.P.)

**Keywords:** COVID-19, ventilation, procalcitonin, survival

## Abstract

Proprotein convertase subtilisin/kexin type 9 (PCSK9) reduces low density lipoprotein (LDL) uptake, leading to increased plasma levels of LDL. In addition, PCSK9 has been implicated in inflammation independently of the effects on cholesterol metabolism. The current analysis showed that our 156 patients with systemic inflammatory response syndrome (SIRS) or sepsis had higher plasma PCSK9 levels in contrast with the 68 healthy controls. COVID-19 sepsis patients had increased plasma PCSK9 levels in comparison to sepsis patients not infected by SARS-CoV-2. For further analysis, patients were divided in two groups based on COVID-19. In both sub-cohorts, plasma PCSK9 levels did not correlate with C-reactive protein, leukocyte count, and procalcitonin. Plasma PCSK9 levels of both patient groups did not significantly differ among SIRS/sepsis patients with and without dialysis and patients with and without ventilation. Furthermore, vasopressor therapy was not significantly associated with altered plasma PCSK9 levels. In the non-COVID-19 SIRS/sepsis group, patients with Gram-negative and Gram-positive infections had similar plasma PCSK9 levels as patients without a detectable pathogen in their blood. In conclusion, the current study suggests PCSK9 as a possible biomarker for COVID-19, but this needs to be validated in larger cohorts.

## 1. Introduction

Sepsis is a very severe disease characterized with a high mortality. A dysregulated immune response with high serum levels of pro- as well as anti-inflammatory cytokines is a hallmark of sepsis [1,2]. Severe acute respiratory syndrome coronavirus type 2 (SARS-CoV-2) infection can cause pneumonia, which may progress to acute respiratory distress syndrome (ARDS). Many critically ill patients with Coronavirus disease 2019 (COVID-19) present with sepsis-related clinical manifestations, including multiple organ dysfunction, coagulopathy, systemic inflammation, and septic shock [3].

Septic patients have reduced levels of serum cholesterol. Low levels of high-density lipoprotein (HDL) as well as low-density lipoprotein (LDL) were found to be associated with an increase in the rates of adverse clinical outcomes and mortality [4,5,6,7]. Cholesterol supplementation in sepsis therapy revealed divergent results in animal studies. Unfortunately, human studies attempting to rise serum cholesterol are limited in availability [8]. Proprotein convertase subtilisin/kexin type 9 (PCSK9) causes degradation of the LDL receptor resulting in a reduced hepatic uptake of LDL along with an accordingly increased serum LDL level. PCSK9 is highly expressed in the liver and its circulating levels decline in patients with liver cirrhosis [9,10,11]. Patients with liver cirrhosis have an increased risk for sepsis-related death [12], but a causal effect of PCSK9 on survival has not been described.

Blockage of PCSK9 by monoclonal antibodies efficiently lowers the serum cholesterol and LDL levels [10]. Considering that serum cholesterol is typically low during severe illness, it might therefore be assumed that PCSK9 is also reduced. Several studies did, however, report higher PCSK9 levels in critical illness [10,12,13,14].

The LDL-receptor-mediated uptake of LDL enables the clearance of bacterial lipids, such as lipopolysaccharides and lipoteichoic acid bound to LDL from the circulation, indicating that PCSK9 contributes to inflammation [15]. Injection of lipopolysaccharide into healthy volunteers with PCSK9 loss-of-function mutations induced less inflammation compared to PCSK9 wild-type controls [16]. Patients with PCSK9 loss-of-function mutations had better rates of survival from septic shock compared to the patients with wild-type alleles.

It has to be noted that some studies failed to show significant associations between circulating PCSK9 levels with sepsis severity [10,17]. The risk to develop sepsis in patients hospitalized for infections was not found to be significantly related to genetic PCSK9 variants [18]. In accordance with this study, a meta-analysis including 20 studies and 64,984 patients showed that treatment with PCSK9-inhibiting antibodies did not modify the risk for severe viral and bacterial infections and sepsis [19]. Randomized clinical trials reporting the benefits of PCSK9 inhibitors on LDL levels did not show a decline in the systemic high-sensitive C-reactive protein (CRP) concentrations [20].

Notably, a single subcutaneous injection of the monoclonal PCSK9 antibody evolocumab reduced the need for intubation and/or mortality and lowered serum interleukin (IL)-6 in patients with severe COVID-19 [21]. During this study, LDL-cholesterol declined in the PCSK9 antibody-treated patients by 14% and increased in the placebo group by 45%, respectively. Circulating levels of PCSK9 were not reported by these authors [21]. Plasma levels of PCSK9 were, however, not found to be associated with death in a cohort of hospitalized COVID-19 patients [22]. In ARDS patients, high PCSK9 levels were not found to be related with mortality [23]. Plasma PCSK9 levels in COVID-19 patients, as well as ARDS patients were about 340 ng/mL, but a comparison with healthy controls was not provided by the authors [22,23]. We are not aware of studies having compared the systemic PCSK9 levels of healthy controls and COVID-19 patients, or COVID-19 patients with and without sepsis.

Common underlying diseases in sepsis include cancer, autoimmune disease, and chronic liver disease [24]. CRP and procalcitonin are frequently used as clinical markers for various infectious diseases [25,26]. Procalcitonin is a suitable parameter for the early diagnosis of bacterial infections [27]. Chronic liver diseases are associated with a change in the circulating levels of CRP and procalcitonin [27,28], thus reducing the diagnostic utility of these surrogate markers in patients with hepatic dysfunction [29].

The aim of our study was to evaluate the diagnostic performance of plasma PCSK9 in systemic inflammatory response syndrome (SIRS)/sepsis patients with COVID-19 disease, and to compare these plasma PCSK9 levels with established laboratory markers of inflammation and sepsis in the context of underlying comorbidities.

## 2. Materials and Methods

### 2.1. Study Cohort

From August 2018 until January 2023, respectively, the plasma of 156 adult patients with different causes of systemic inflammatory response syndrome (SIRS, 27 patients), sepsis (40 patients), and septic shock (79 patients) was collected at the University Hospital of Regensburg. Plasma of the 23 patients infected with SARS-CoV-2 was collected from October 2020 until January 2023, respectively. All of the COVID-19 patients had sepsis due to suffering from viral infections. Co-infection with Gram-negative bacteria occurred in 3 COVID-19 patients, co-infection with Gram-positive bacteria in 5 COVID-19 patients, and co-infection with Gram-negative as well as Gram-positive bacteria in 3 COVID-19 patients, respectively. The bacterial infection rates did not differ between the COVID-19 and non-COVID-19 patients in accordance with a recent study [30].

The Sepsis-3 criteria were used for the categorization of these patients [31]. Patients who were admitted to the intensive care unit with suspected sepsis but did not develop sepsis during their follow-up were categorized as SIRS, as they fulfilled the SIRS criteria [32]. Patients with multi-resistant infections, viral hepatitis, or human immunodeficiency virus infection were excluded. Laboratory values were obtained from the Institute of Clinical Chemistry and Laboratory Medicine located at our University Hospital.

The control group consisted of 40 males and 28 females, respectively. These healthy controls were students, employees of our university hospital, or relatives of the employees. Age of the controls was 55 (21–80) years. Gender distribution and age of the patients with SIRS/sepsis and healthy controls were found to be comparable (*p* > 0.05). Age and gender distribution of the controls and the COVID-19 patients was found to be similar (*p* > 0.05).

### 2.2. PCSK9 ELISA

Blood samples were collected from 12 to 24 h after the admission of the patients to the intensive care unit. EDTA was used as the anticoagulant, and the plasma was subsequently prepared. The human PCSK9 DuoSet ELISA (R&D Systems; Wiesbaden, Nordenstadt, Germany) was used as suggested by the provider (the dilution of plasma was 1:100). The capture antibody was diluted in phosphate-buffered saline (PBS) and was then used to coat a 96-well microplate. The sealed plate was incubated overnight at room temperature. The capture antibody was removed the following morning and the microplate was washed three times with PBS. Blocking was achieved by incubation with reagent dilution solution for 1 h at room temperature. After 3 times washing, 100 µL of the diluted plasma was added to the wells. A seven-point standard curve ranging from 125 pg/mL to 8000 pg/mL, respectively, was prepared and processed in parallel with the samples. Each standard sample and each blood sample was analyzed in duplicate and for calculations the mean values were used. The covered microplate was incubated for 2 h at room temperature. After washing for three times the detection antibody was added, and the microplate was incubated for 2 h at room temperature and washed again. Then, the streptavidin-horse radish peroxidase solution was added for 20 min. After washing, the substrate solution was added and then incubated for 20 min in the dark. After adding the stop solution the optical density was measured at 450 nm. The wave length correction was set to 540 nm. The values of the blanks, which were wells treated identically as all other wells but without the addition of plasma, were subsequently subtracted.

### 2.3. Statistical Analysis

Boxplots graphically demonstrate the minimum and the maximum values, the median, and the first and third quartiles. Outliers are plotted as individual circles or asterisks. The median, minimum, and maximum values are listed in the tables. Data were analyzed using the non-parametric Mann–Whitney U test, the Kruskal–Wallis test, and Spearman’s correlation (IBM SPSS Statistics 26.0 program). Categorial variables were compared using the chi-squared test (Ms Excel, version 2003). A value of *p* < 0.05 was considered statistically significant.

## 3. Results

### 3.1. PCSK9 in Controls, SIRS/Sepsis Patients, and SIRS/Sepsis Patients with Liver Cirrhosis

**Figure 1:**
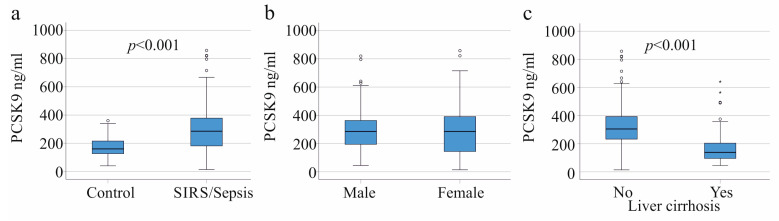
PCSK9 in the plasma of the controls, SIRS/sepsis patients, and SIRS/sepsis patients with and without liver cirrhosis. (**a**) Plasma PCSK9 levels of the 68 controls and the 156 SIRS/sepsis patients; (**b**) Plasma PCSK9 of the 109 male and 47 female SIRS/sepsis patients. Plasma PCSK9 levels across males and females were similar; (**c**) Plasma PCSK9 of 32 SIRS/sepsis patients with (Yes) and 124 SIRS/sepsis patients without liver cirrhosis (No). Outliers are plotted as individual circles or asterisks.

The plasma PCSK9 concentration of the 156 patients with SIRS or sepsis was 285 (14–858) ng/mL and was found to be significantly higher in comparison to the 68 controls with 160 (40–360) ng/mL (Figure 1a). In the control cohort as well as the SIRS/sepsis cohort, men and women were found to have similar plasma PCSK9 levels (*p* = 0.732 and *p* = 0.337, respectively) (Figure 1b). However, PCSK9 in plasma did not correlate with age, and the correlation coefficient r was—0.194 (*p* = 0.271) for the controls and was 0.022 (*p* = 0.781) for the SIRS/sepsis group, respectively. PCSK9 has been described to decline in patients with liver cirrhosis [11], and this was also observed in the SIRS/sepsis cohort studied herein (Figure 1c).

Comparison of the entire study cohort with the subgroup of patients with liver cirrhosis showed that the subgroup of patients with liver cirrhosis had low levels of the C-reactive protein (CRP) in comparison with the whole cohort. Age, gender distribution, procalcitonin, and leukocyte count did not significantly differ between these groups (Table 1).

### 3.2. PCSK9 in SIRS/Sepsis Patients without Liver Cirrhosis Stratified for Underlying Diseases and Infectious Diseases

**Figure 2:**
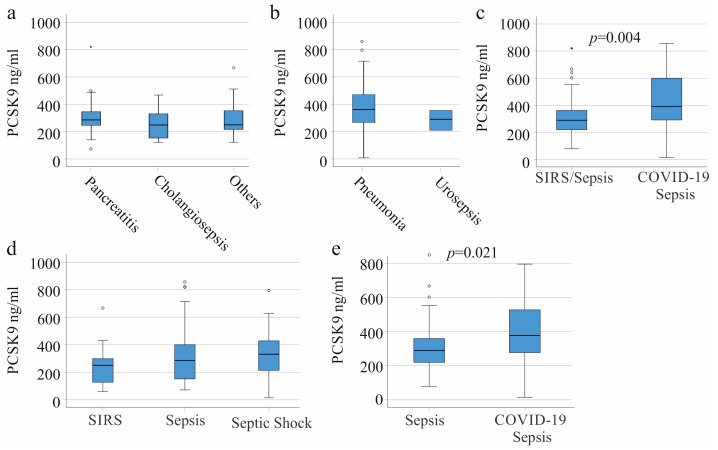
PCSK9 in the plasma of patients with SIRS/sepsis stratified for underlying diseases and causes of inflammation and sepsis (patients with liver cirrhosis were excluded). (**a**) Plasma PCSK9 levels of SIRS/sepsis patients with different underlying diseases. Pancreatitis (31 patients), cholangiosepsis (9 patients), and others (29 patients); (**b**) Plasma PCSK9 levels of SIRS/sepsis patients with different causes of inflammation. Pneumonia (41 patients) and urinary tract infections (14 patients); (**c**) Plasma PCSK9 of 103 SIRS/sepsis patients without and 21 patients with SARS-CoV-2 infection; (**d**) Plasma PCSK9 of patients categorized according to the SIRS criteria and to the Sepsis-3 definition as SIRS (27 patients), sepsis (33 patients), and septic shock (64 patients); (**e**) Plasma PCSK9 of 76 sepsis patients without and 21 sepsis patients with SARS-CoV-2 infection (patients with SIRS were excluded). The *p*-values are shown in the figures when significant differences between the groups were observed. Outliers are plotted as individual circles or asterisks.

Along with liver cirrhosis (32 patients), the most common underlying diseases observed with these patients were pancreatitis (31 patients) and cholangiosepsis (9 patients). Other underlying diseases, such as cancer, were rare, and these patients were integrated in one group (29 patients). Plasma PCSK9 levels did not significantly differ between these groups of patients (Figure 2a).

Common infections that lead to sepsis were pulmonary (41 patients) and urinary tract infections (14 patients). Plasma PCSK9 levels were found to be similar between these groups of patients (Figure 2b).

Of clinical relevance, the 21 COVID-19 patients with sepsis had higher plasma PCSK9 levels in comparison with the SIRS/sepsis patients not infected with SARS-CoV-2 (Figure 2c). CRP and leukocyte count were similar between these two groups, whereas procalcitonin levels of the COVID-19 patients were low (Table 1). Even in the subgroup of patients super-infected with bacteria, COVID-19 patients tended to have low procalcitonin levels. Procalcitonin was 3.2 (0.05–270.0) ng/mL in SIRS/sepsis patients with bacterial infections and was 0.58 (0.15–65.4) ng/mL in sepsis patients with bacterial and SARS-CoV-2 coinfection, respectively (*p* = 0.113).

The patients were categorized as SIRS, sepsis, and septic shock [31]. PCSK9 levels in plasma appeared to be higher in septic shock and sepsis in comparison to SIRS (*p* = 0.139; Figure 2d). The COVID-19 cohort did not include patients with SIRS (Table 1). The exclusion of patients with SIRS from this cohort showed that plasma PCSK9 was still higher in COVID-19 patients (Figure 2e).

A substantial variability in plasma PCSK9 levels was observed among the COVID-19 and non-COVID-19 patients. The overlap in the plasma levels of PCSK9 between the COVID-19 patients and the non-COVID-19 patients was nearly 95% (Figure 2c,e). In the subgroup of patients with liver cirrhosis, only two patients had COVID-19, and associations of PCSK9 levels with viral infection could not be analyzed.

### 3.3. Plasma PCSK9 in Relation to Vasopressor Therapy and Interventions

The associations of plasma PCSK9 with the need for dialysis, ventilation, or catecholamine treatment were calculated for the SIRS/sepsis patients (1) without liver cirrhosis and COVID-19, (2) patients with liver cirrhosis after the exclusion of patients with COVID-19, and (3) patients with COVID-19 after the exclusion of patients with liver cirrhosis (Table 2).

Plasma PCSK9 was not found to be significantly related to the need for dialysis or ventilation in any of these subgroups. Patients with liver cirrhosis under vasopressor therapy had 162.4 (44.7–641.2) ng/mL, while those without had 111.3 (59.0–173.6) ng/mL PCSK9 in plasma, and this difference was determined to be significant (Table 2).

### 3.4. Plasma PCSK9 in Relation to Inflammation Markers

In the entire cohort, a significantly positive correlation between plasma PCSK9 and CRP was identified (r = 0.304, *p* < 0.001). Associations with the leukocyte count or procalcitonin were not detected. When patients with COVID-19 and liver cirrhosis were excluded, plasma PCSK9 did not correlate with the CRP, leukocyte count, or procalcitonin (Table 3). In the subgroup of COVID-19 patients, plasma PCSK9 was not found to be significantly associated with any of these established clinical markers of inflammation (Table 3). Patients with liver cirrhosis had low CRP levels in comparison with the other subgroups (Table 1). Leukocyte count and procalcitonin were similar between these groups (Table 1). Notably, plasma PCSK9 positively correlated with the leukocyte count, procalcitonin, and CRP in patients with SIRS/sepsis and liver cirrhosis (Table 3).

IL-6 and ferritin are known biomarkers for COVID-19 severity [33]. The IL-6 concentration was 74.1 (15.7–8679.0) pg/mL, and the ferritin levels were 1193 (200–17,846) ng/mL in the COVID-19 cohort. Of note, the levels of these markers did not significantly correlate with plasma PCSK9 (Table 3).

### 3.5. Plasma PCSK9 in Gram-Negative and Gram-Positive Infection

**Figure 3:**
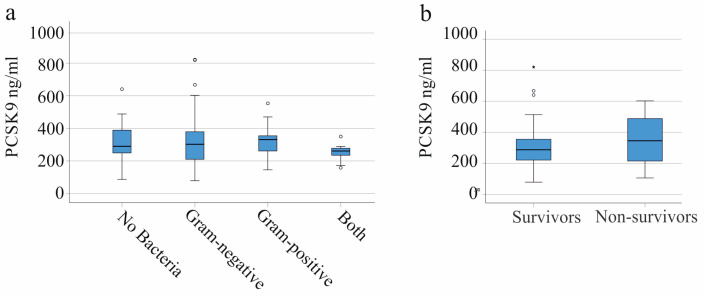
PCSK9 in the plasma of patients with SIRS/sepsis (patients with COVID-19 and patients with liver cirrhosis were excluded) stratified for the type of bacterial infection and the association of plasma PCSK9 with survival. (**a**) Plasma PCSK9 levels stratified for the type of bacterial infection (thirty-four patients had no detectable bacterial pathogen, forty-nine patients had Gram-negative infection, nine patients had Gram-positive infection, and nine patients had both Gram-negative and Gram-positive bacteria). (**b**) Plasma PCSK9 levels of the 84 SIRS/sepsis patients who survived and the 18 patients who did not survive. There were no significant differences observed between these groups. Outliers are plotted as individual circles or asterisks.

For this analysis, patients with liver cirrhosis and patients with COVID-19 were excluded. The plasma PCSK9 levels of the thirty-four patients with no bacterial infectious agents detectable was found to be comparable with the levels of the forty-nine Gram-negative infected patients, the nine patients with Gram-positive bacteria, and the eleven patients with Gram-negative and Gram-positive bacteria in their blood cultures (Figure 3a). In the COVID-19 sub-cohort, eleven patients were co-infected with bacteria (three Gram-negative, five Gram-positive, and three with Gram-negative and Gram-positive bacteria, respectively). Plasma PCSK9 levels did not significantly differ between patients with and without bacterial co-infections (*p* = 0.557).

### 3.6. Plasma PCSK9 and Survival

Plasma PCSK9 was not found to be significantly related with survival. PCSK9 in the plasma of 84 patients (without patients with COVID-19 and patients with liver cirrhosis) who survived and 18 patients who died was determined to be similar (*p* = 0.243) (Figure 3b).

In the COVID-19 cohort (without patients with liver cirrhosis), seven patients died, and their PCSK9 levels did not significantly differ from the patients who survived (*p* = 0.128). Eleven patients with liver cirrhosis died (without patients with COVID-19), and their plasma PCSK9 levels was also comparable to the patients who survived (*p* = 0.497).

## 4. Discussion

This study showed that plasma PCSK9 is induced in SIRS/sepsis patients in contrast to the controls. Septic COVID-19 patients had the highest plasma PCSK9 levels, meaning therefore that systemic PCSK9 may become a novel biomarker for SARS-CoV-2 infection.

PCSK9 is a well-known regulator of plasma LDL levels, and antibodies against PCSK9 are used for the therapy of hypercholesterinemia [34]. Severe diseases such as sepsis are associated with hypocholesterinemia, a relevant prognostic factor for mortality [8]. In accordance with the current findings, several studies have reported that PCSK9 levels are increased in sepsis [10,35]. However, the described positive association of PCSK9 and serum cholesterol [10] is not found in sepsis.

It has been reported that an inappropriate induction of PCSK9 upon bacterial infection was associated with mortality [13]. However, other investigations do not support a role of PCSK9 in sepsis. These studies did not observe associations between PCSK9 expression and the risk of sepsis or beneficial effects of PCSK9-blocking antibodies in sepsis [18,19].

Discordant findings may partly be due to the inhomogeneous study cohorts. Patients with liver cirrhosis have a higher risk for infections and sepsis [12], and the plasma PCSK9 level of these patients has been described as low [11,36]. The association of PCSK9 levels with survival described in the study cited above was no longer significant when patients with chronic liver diseases were excluded [24]. Therefore, it is recommendable to calculate associations of PCSK9 with disease severity and/or mortality in sepsis patients following the exclusion of patients with severe chronic liver diseases.

Moreover, to our knowledge, this is the first study showing that COVID-19 sepsis patients have higher plasma PCSK9 levels compared to healthy controls and in comparison with sepsis patients not infected with SARS-CoV-2. The PCSK9 expression of peripheral blood mononuclear cells was found to be induced in COVID-19 patients [36]. The cell type which contributes to plasma PCSK9 in COVID-19 is unknown as of yet. Our results, in agreement with the beneficial effects of inhibitory PCSK9 antibody therapy in COVID-19 [21], indicate harmful effects of PCSK9 in COVID-19.

Importantly, PCSK9 levels of COVID-19 patients were not found to be significantly correlated with the CRP, procalcitonin, IL-6, or ferritin. This illustrates that in COVID-19 PCSK9 is an independently regulated biomarker.

Plasma PCSK9 levels did not differ significantly among the patients with and without dialysis, ventilation, or vasopressor therapy. The exceptions observed were patients with liver cirrhosis on vasopressor therapy, who had higher plasma PCSK9 levels. Vasopressor therapy was not related to altered PCSK9 levels in the remaining patient cohort, and more research is therefore needed to explain this sub-cohort-specific effect.

PCSK9 plasma levels were not related with survival. This is in accordance with the findings that were obtained in ARDS patients [23]. However, other studies have shown that patients with PCSK9 loss-of-function mutations had better rates of survival in septic shock [16] and Gram-positive sepsis [37]. Although this latter finding may suggest that systemic PCSK9 levels are induced in Gram-positive sepsis [37], our study showed comparable plasma PCSK9 levels of patients with Gram-negative and Gram-positive infections. Patients infected with bacteria had similar plasma PCSK9 levels as patients who had no detectable bacteria in their blood cultures. This principally argues against systemic PCSK9 levels as a marker of bacterial infections. An equal rise in plasma PCSK9 in Gram-negative and Gram-positive blood infections has been shown before [13], and therefore, higher PCSK9 levels may be a marker of severe illness rather than for bacterial infections. Our data characterizes PCSK9 as a possible, clinically relevant biomarker for viral infections, and this should be further evaluated in multi-center studies.

The improved clearance of pathogenic toxic lipids via the LDL receptor was supposed to dampen the inflammatory response [16]. The current evidence does not allow a final conclusion on the role of PCSK9 in severe illness. Furthermore, the expression of the LDL receptor, circulating LDL levels, or its comorbidities have to be considered when calculating associations between the PCSK9 levels and mortality.

Procalcitonin is a pro-hormone released by the thyroid parafollicular cells, and is induced in all parenchymal cells in the case of microbial infections [38]. Plasma procalcitonin was found to be low in COVID-19 sepsis. Even in patients with viral and bacterial coinfections, the levels of procalcitonin were also determined to be low. This finding further suggests that the value of biomarkers has to be assessed with the clinical context [25,38].

In line with this assumption, positive correlations between plasma PCSK9 and the markers of inflammation, namely CRP, leukocyte count, and procalcitonin were only detected in patients with liver cirrhosis. In the COVID-19 cohort and in the sepsis cohort, after patients with liver cirrhosis were excluded, no such associations were observed. Even though there is currently no explanation for this finding, these results further point to the disease-specific associations between PCSK9 and inflammation. Notably, plasma PCSK9 did not correlate with IL-6 and ferritin, which were shown before to be related to disease severity in SARS-CoV-2 infection [33]. This finding suggests that PCSK9 could become an early biomarker for COVID-19 sepsis.

The present evidence indicates that the circulating levels as well as the associations of PCSK9 with the clinical markers of inflammation are affected by liver cirrhosis and SARS-CoV-2 infection. Underlying diseases, such as pancreatitis and cholangitis, were, however, not related to altered systemic PCSK9 levels.

Age and gender may be confounding factors in observational studies. PCSK9 levels were similar between sexes and were not associated with age in the cohort studied herein. Higher circulating levels of PCSK9 in females have been reported [39], whereas other studies did not detect a gender difference [11,40]. Modest negative as well as positive associations of serum PCSK9 with age have been observed in a few studies, whereas age was not associated with circulating PCSK9 levels in other cohorts [11,24,41].

Our study has several limitations. First, this was a single center study, and most patients originated from Germany, meaning these results may not be valid for other ethnicities. The sub-cohorts with SARS-CoV-2 infection and patients with liver cirrhosis were very small, and this greatly limited the statistical power as a result. Overlap in the PCSK9 levels of the COVID-19 patients and the non-COVID-19 patients made it difficult to establish the cut-off values, and therefore, studies on larger cohorts are needed. Substantial variability in the plasma PCSK9 levels was observed among these patients, and the identification of factors influencing plasma PCSK9 during severe illness is a challenge for the future.

## 5. Conclusions

The current study shows that SIRS/sepsis patients have higher PCSK9 levels in comparison to healthy controls. Plasma PCSK9 further increased in COVID-19 sepsis. To our knowledge this is the first study showing that PCSK9 may possibly become a biomarker for COVID-19 sepsis, or a biomarker for viral infections in general. Future research in larger cohorts is needed to evaluate the clinical utility of PCSK9.

## Figures and Tables

**Table 1:** Characteristics of the SIRS/sepsis patients. Data are given for the whole cohort, for SIRS/sepsis patients without liver cirrhosis, for patients with liver cirrhosis (without COVID-19 patients), and for patients with COVID-19 (liver cirrhosis patients were excluded). The subgroups were compared to the whole study group and variables, and those which differed significantly between two groups were marked with the identical symbols. The respective *p*-values are * *p* < 0.05, *** *p* < 0.001, ^§§§^ *p* < 0.001.

Parameter	All Patients	Patients with Liver Cirrhosis Excluded	Patients with Liver Cirrhosis (COVID-19 Patients Excluded)	COVID-19 Patients (Patients with Liver Cirrhosis Excluded)
Males/Females	109/47	86/38	22/8	15/6
Age (years)	59 (21–93)	57 (21–88)	58 (31–75)	63 (29–80)
SIRS/Sepsis/Septic shock	37/40/79	27/33/64	10/7/15	00/2/19
C-reactive protein mg/L	157 (12–697) *** ^§§§^	183 (35–597) ***	61 (12–236) ^§§§^	156 (44–472)
Procalcitonin ng/mL	1.15 (0.05–270) *	1.17 (0.06–270.00)	1.17 (0.10–65.18)	0.57 (0.08–65.40) *
Leukocytes n × 10^9^/L	10.31 (0.06–1586.00)	10.35 (2.16–37.38)	10.95 (2.51–1586.00)	9.62 (2.78–18.47)

**Table 2:** Plasma PCSK9 (ng/mL) levels of patients with and without dialysis, ventilation, and vasopressor therapy. The number of patients treated is given in “N” and the respective *p*-values are listed.

Intervention/Drug	Patients without Liver Cirrhosis and without COVID-19 (103)		Patients with Liver Cirrhosis and without COVID-19 (30)		Patients with COVID-19 without Liver Cirrhosis (21)	
	N	*p*-Value	N	*p*-Value	N	*p*-Value
Dialysis	29	0.538	14	0.552	9	0.651
Ventilation	54	0.160	18	0.124	21	-
Vasopressor therapy	55	0.500	19	0.030	19	0.467

**Table 3:** Correlation coefficient (r) and *p*-values for the correlation of plasma PCSK9 with the clinical markers of inflammation. Number of patients per subgroup are given in brackets. IL-6 and ferritin were only measured in the COVID-19 patients.

Biomarker of Inflammation	Patients without Liver Cirrhosis without COVID-19 (103)		Patients with Liver Cirrhosis without COVID-19 (30)		Patients with COVID-19 without Liver Cirrhosis (21)	
	r	*p*-Value	r	*p*-Value	r	*p*-Value
Leukocyte count	0.127	0.200	0.404	0.027	0.143	0.537
Procalcitonin	−0.016	0.871	0.405	0.027	−0.182	0.430
C-reactive protein	0.153	0.124	0.603	<0.001	−0.171	0.457
IL-6					0.271	0.234
Ferritin					0.038	0.871

## Data Availability

Data supporting reported results can be obtained from the corresponding author.
